# Bacterial Changes in Boiled Crayfish between Different Storage Periods and Characterizations of the Specific Spoilage Bacteria

**DOI:** 10.3390/foods12163006

**Published:** 2023-08-09

**Authors:** Jiangyue Xia, Ning Jiang, Bin Zhang, Rongxue Sun, Yongzhi Zhu, Weicheng Xu, Cheng Wang, Qianyuan Liu, Yanhong Ma

**Affiliations:** 1 Zhejiang Provincial Key Laboratory of Health Risk Factors for Seafood, College of Food and Pharmacy, Zhejiang Ocean University, Zhoushan 316022, China; xiajiangyue9@163.com; 2 Institute of Agricultural Products Processing, Jiangsu Academy of Agricultural Sciences, Nanjing 210014, China; sunrongxue187@163.com (R.S.); 19970030@jaas.ac.cn (Y.Z.); 212748027@njnu.edu.cn (W.X.); wangcheng@jaas.ac.cn (C.W.); liu.qianyuan@foxmail.com (Q.L.); 20090008@jaas.ac.cn (Y.M.); 3 Integrated Scientific Research Base for Preservation, Storage and Processing Technology of Aquatic Products of the Ministry of Agriculture and Rural Affairs, Nanjing 210014, China

**Keywords:** crayfish, bacterial community, spoilage potential, high-throughput sequencing

## Abstract

This study investigated changes in the microbial compositions of crayfish tails during storage at 4 °C (for 0–12 days) as measured using high-throughput sequencing (HTS). The specific spoilage organisms (SSOs) in the crayfish tails were isolated using culture-dependent cultivation methods, and they were identified by 16S rRNA and characterized for their enzymatic spoilage potentials (e.g., protease, lipase, phospholipase, and amylase). The spoilage abilities of the selected strains in the crayfish tails were assessed by inoculating them into real food. Moreover, the microbial growth and the volatile basic nitrogen (TVB-N) changes were monitored during the storage period. The results from the HTS showed that the dominant genus of shrimp tails evolved from *Streptococcus* (D0) to *Pseudomonas* (D4) and, finally, to *Paenisporosarcina* (D12) during storage. Seven bacterial species (*Acinetobacter lwoffii, Aeromonas veronii, Kurthia gibsonii, Pseudomonas sp.*, *Exiguobacterium aurantiacum*, *Lelliottia amnigena,* and *Citrobacter freundii*) were screened from the spoiled shrimp tails by the culture-dependent method, among which *Aeromonas veronii* had the strongest spoilage ability.

## 1. Introduction

Crayfish (*Procambarus clarkii*) are one of the most important economic aquatic products in China. Due to their tender texture, delicate taste, and high protein content, crayfish are gaining increased popularity among customers. Currently, crayfish are typically farmed in the interior of China and are widely distributed throughout the country [1]. The high water content and free amino acid content of crayfish make them susceptible to spoilage by oxidation reactions [2], bacteria, and endogenous enzymes [3]. In recent years, bacterial spoilage has become a widespread concern in industries, with a focus on the dynamics of spoilage bacteria during storage. Indeed, changes in bacterial community structures may cause different types of spoilage defects or affect the shelf life of food [4]. Therefore, monitoring the development of bacterial communities during food storage and the original microbiota of raw materials could significantly be of benefit for identifying the predominant spoilage bacteria, preventing spoilage, and extending the shelf-lives of food products [5].

Culture-dependent bacterial cultivation and characterization methods and culture-independent methods have been widely used to analyze bacteria in food products. Culture-dependent methods mainly involve culturing and screening different strains on various selective media. Single colonies of selected strains are isolated to obtain pure cultures, and then genomic DNA are extracted from the pure cultures. Afterwards, molecular analyses are performed on the extracted DNA using polymerase chain reaction (PCR) to determine the strain species. Culture-dependent methods have been widely used to screen and identify spoilage bacteria in spoiled chicken [6], bacon [7], and shrimp [8] products. The limitation of culture-dependent methods is that not all the bacteria present in a product can be cultured on the media for the bacterial analyses [9], and only 0.1–3.0% of the bacteria can be cultured, which may not represent all the bacteria in a product [10].

With the developments in molecular biology, molecular techniques such as polymerase chain reaction and denaturing gradient gel electrophoresis (PCR-DGGE), terminal restriction fragment length polymorphism (T-RFLP), real-time PCR, and conventional PCR and other technologies have been used to investigate the compositions of bacterial communities in aquatic products [11,12,13,14]. However, all of these approaches are usually laborious and expensive, and they require high bioinformatic skills for raw data analyses [15] Compared with other molecular techniques, high-throughput sequencing (HTS) is a powerful technology that can be used to gain insight into the bacterial changes in food. HTS has been widely used to analyze the changes in the bacterial communities in food products during storage, including crabs [16], carp [17], clams [18], and other aquatic products. In contrast, HTS provides a more advanced approach that can help scientists to assess and understand bacteria, and it provides a comprehensive description of the complex interactions among species [19]. Because HTS can obtain more reads than culture-independent methods, it can help to discover more bacterial diversity and detect large amounts of bacteria of unknown origin [20]. Consequently, HTS is more effective in monitoring the evolution of bacterial communities in products during storage. Therefore, an approach that combines culture-dependent and culture-independent methods could be appropriate for investigating bacterial diversity and characterizing the specific spoilage organisms (SSOs) in food. However, to the best of our knowledge, the microbial community of boiled crayfish during refrigerated storage has not been studied using a combination of HTS and traditional cultivation methods. 

Therefore, this study investigated the microbial communities in boiled crayfish using a combination of culture-dependent and culture-independent methods. The diversity and dynamics of the microbial communities in boiled crayfish were analyzed during refrigerated storage. Further, the SSOs of the boiled crayfish tails were isolated and characterized for their spoilage potentials. The results obtained in this study will be used to exploit appropriate control strategies for the preservation of crayfish products.

## 2. Materials and Methods

### 2.1. Sample Preparation

#### 2.1.1. Boiled Crayfish Tails

Crayfish weighing 22.0 ± 2.0 g and measuring 13 ± 2 cm in length were collected from the Hongze Lake aquaculture area in Huaian of Jiangsu Province, China. The live crayfish were packed in insulated polystyrene boxes with ice and transported to the laboratory. Upon arrival, all crayfish were washed thoroughly with running water, and the crayfish tails were obtained by twisting the abdomen from the cephalothorax. 

Raw crayfish tails were placed in stainless steel pots filled with boiling water (50 tails per 1 L of water) and cooked for five minutes in an induction heating cooker (C21-IH50E, 2100 W, SUPOR, Zhejiang, China). After boiling, the crayfish tails were removed, drained for 2 min, packaged in sterile polyethylene bags, and stored at 4 °C for 12 days. The total viable counts (TVC) and total volatile basic nitrogen (TVB-N) were measured every two days.

#### 2.1.2. Inoculation

All strains inoculated in this study were previously isolated from boiled crayfish tails at the end of their shelf-lives (on day 12). Each strain was mixed with 30 mL of tryptic soy broth (TSB, Hope Bio-Technology Co., Ltd., Qingdao, China) in a centrifugal tube and cultured in a concussion incubator (HZQ-F100, TC-huamei, Taicang, Jiangsu, China) at 37 °C for 20–24 h to achieve a level of approximately 8.0–9.0 log cfu/mL. Then, the medium was centrifuged at 5000× *g* for 10 min and collected in a 50 mL centrifugal tube to remove the medium. The precipitate was finally collected and washed twice with 30 mL of sterile saline solution (0.85% NaCl). The bacterium suspension was pooled in a sterile beaker and diluted in sterile saline solution in order to achieve an inoculation mixture containing 6.0–7.0 log cfu/mL. The sterile crayfish tails were soaked in each bacterium inoculation mixture (50 tails per 1 L of mixture) for 30 min. After the inoculation, the inoculated crayfish tails were randomly packaged in sterile polyethylene bags and stored at 4 °C for 12 days. 

### 2.2. Microbiological Analysis

The tail meat was removed from the shell aseptically on a clean bench. From each sample, an aliquot (10 ± 1 g) of each sample was transferred to sterile stomacher bags and homogenized with Peptone Physiological Solution (PPS) (1 g/L peptone (Sinopharm Group Co., Ltd., Shanghai, China) plus 8.5 g/L NaCl (Sinopharm)) with a microbiological homogenizer (XO-6D, 220 W, Xianou, Nanjing, China). Then, the mixtures were serially diluted (1:10) in PPS. Afterward, 0.1 mL of the dilutions were spread-plated on Plate Count Agar (Aoboxing Universeen Bio-Technology Co., Ltd., Beijing, China) and incubated at 37 ± 1 °C for 48 h to calculate the TVC.

### 2.3. Measurement of the TVB-N

The TVB-N values of the samples were determined according to the method of Conway and Byrne [21]. From each sample, five grams were dispersed in 50 mL of distilled water and defibrated at high speed for 60 s. The mixture was then run through filter paper and collected. The TVB-N contents were analyzed using a micro diffusion method. The TVB-N values ere expressed as mg TVB-N per 100 g of muscle.

### 2.4. Culture-Independent Methods

#### 2.4.1. DNA Extraction and PCR Amplification

HTS was performed on the raw crayfish tail meat and the boiled crayfish tail meat at days 0, 4, and 12 of storage. The total DNA of the microorganisms was extracted with a TGuide S96 Magnetic Soil/Stool DNA kit (Tiangen Bio-Technology Co., Ltd. Beijing, China) according to manufacturer’s instructions. The DNA concentrations of the samples were measured with a Qubit dsDNA HS assay kit and a Qubit 4.0 fluorometer (Thermo Fisher Scientific, Waltham, MA, USA). The tag-encoded HTS was carried out using an Illumina HiSeq platform (Biomarker Technologies Co., Ltd., Beijing, China).

The 338F (5′-ACTCCTACGGGAGGCAGCA-3′) and 806R (5′-GGACTACHVGGGTWTCTAAT-3′) universal primer set was used to amplify the V3-V4 region of the 16S rRNA gene from the genomic DNA extracted from each sample. Both the forward and reverse 16S primers were tailed with sample-specific Illumina index sequences to allow for deep sequencing. The PCR was performed in a total reaction volume of 10 μL of the following: DNA template 5–50 ng, *Vn F (10 μM) 0.3 μL, *Vn R (10 μM) 0.3 μL, KOD FX Neo Buffer 5 μL, dNTP (2 mM each) 2 μL, KOD FX Neo 0.2 μL, and ddH2O up to 10 μL. The Vn F and Vn R were selected according to the amplification area. The initial denaturation at 95 °C for 5 min was followed by 25 cycles of denaturation at 95 °C for 30 s, annealing at 50 °C for 30 s, and extension at 72 °C for 40 s, with a final step at 72 °C for 7 min. The total PCR amplicons were purified with Agencourt AMPure XP beads (Beckman Coulter, Indianapolis, IN, USA) and quantified using a Qubit dsDNA HS assay kit and a Qubit 4.0 fluorometer. After the individual quantification step, the amplicons were pooled in equal amounts. For the constructed library, the study used Illumina novaseq 6000 (Illumina, Santiago, CA, USA) for the sequencing.

#### 2.4.2. Data Processing and Taxonomic Classification

According to the quality of a single nucleotide, the raw data were primarily filtered by Trimmomatic (version 0.33) [22]. Identification and removal of the primer sequences were processed by Cutadapt (version 1.9.1) [23]. The PE reads obtained from the previous steps were assembled by USEARCH (version 10) [24], followed by chimera removal using UCHIME (version 8.1) [25]. The high-quality reads generated from above steps were used in the analysis that followed. Sequences with a similarity of ≥97% were clustered into the same operational taxonomic units (OTUs) by USEARCH (version 10.0) [22], and the OTUs with reabundances of <0.005% were filtered. The taxonomy annotations of the OTUs were performed based on the Naive Bayes classifier in QIIME2 using the SILVA database [25], with a confidence threshold of 70%.

The alpha diversity (Goods’ coverage, Chao1 richness, Simpson richness, and Shannon diversity) were calculated and displayed by QIIME2 and R software, respectively. The beta diversity (UPGMA cluster analysis) was determined to evaluate the degree of similarity between the bacterial communities from the different samples using QIIME.

### 2.5. Culture-Dependent Methods

#### 2.5.1. Microbial Enumeration and Bacteria Isolation

A sample of 10 ± 1 g from the boiled crayfish tail meat on day 12 of storage was aseptically transferred to a stomacher bag with 90 mL of PPS and homogenized for 120 s. Volumes of 0.1 mL from 10-fold serial dilutions were spread on the surface of the following media: PCA, incubated at 37 ± 1 °C for 48 h to achieve the TVC and at 7 °C for 10 d for the total psychrotrophic count (TPC); Centrimide-Fucidin-Cepha Loridine (CFC) media (Hopebio) for yielding *pseudomonads*, incubated at 25 ± 1 °C for 48 h; and Rose Bengal (RB, Hopebio) for yeasts and molds, incubated at 28 ± 1 °C for 5 d. The dilutions (1 mL) were used for the analysis of the *Enterobacteriaceae* and lactic acid bacteria (LAB) counts of the samples by Violet Red Bile Glucose Agar (VRBGA, Hopebio) and De Man, Rogosa, and Sharpe (MRS) agar (Hopebio), and they were inoculated at 37 ± 1 °C for 24 h and 37 ± 1 °C for 48 h, respectively. The bacterial colony counts were expressed as log cfu/g.

After incubation, the bacterial colonies were selected according to their different appearances, sizes, shapes, and colors. Then, the isolated colonies were purified on LB Nutrient Agar (Hopebio) at least four times. The isolated bacteria were grown in TSB and stored in 50% (*v*/*v*) sterile glycerol at −20 °C for use in the subsequent analyses.

#### 2.5.2. Screening for the Enzyme Production

The activities of the proteolysis, lipolysis, phosphololysis, and amylolytic enzymes of all the isolated strains were measured by agar diffusion assays at 37 ± 1 °C. Proteolytic enzyme production was determined using skim milk agar (1 × Trypticase Soy Agar (TSA, Hopebio) plus 2% skim milk power). After inoculation, cleaning zones appeared around the bacterial colonies. Tween 80 medium agar (1 × LB agar plus 1% Tween-80 plus 0.1 g CaCl_2_) and egg-yolk agar (1 × LB agar plus 1% egg-yolk emulsion) were used to determine the lipolytic and phosphorolytic enzyme production. At the end of the incubation period of the bacteria, a white opaque zone of precipitation spread beyond the edge of the colony. To test the amylolytic activities of the bacteria, starch agar (1 × LB agar plus 1% starch) was used. When the bacterial incubation was over, a solution of potassium iodide was sprayed on the surfaces of the petri dishes and a colorless transparent zone formed around the bacterial colonies [26,27].

#### 2.5.3. DNA Extraction and Genomic Sequence Amplification

Genomic DNA from the pure cultures was extracted by a Tsingke Bacteria DNA kit (Tsingke Bio-Technology Co., Beijing, China) according to the manufacturer’s instructions. A pair of universal primers, 27F (5′-AGAGTTTGATCCTGGCTCAG-3′) and 1492R (5′-AAGGAGGTGATCCAGCCGCA-3′), were used in amplifying the 16S rRNA gene fragment by PCR. Tsingke (Beijing, China) was in charge of sequencing the purified PCR products. The 16S rRNA gene sequences were compared with known sequences using the BLAST function in the National Center Biotechnology Information (NCBI, http://blast.ncbi.nlm.nih.gov/Blast.cgi, accessed on 9 April 2023). Based on the species identification protocol, sequences that matched with more than 97% similarity were considered to be the same species [28].

### 2.6. Statistical Analysis

The data were analyzed by analysis of variance (ANOVA) using SPSS 22.0 software (SPSS Inc., Chicago, IL, USA). The least significant difference (LSD) procedure was used to determine the significance of the differences, and the means with different letters were considered significant at *p* < 0.05. The results are presented as the means ± standard deviations (SDs) of triplicate measurements of the replicates.

## 3. Results and Discussion

### 3.1. Changes in TVC and TVB-N with Storage Time

**Figure 1:**
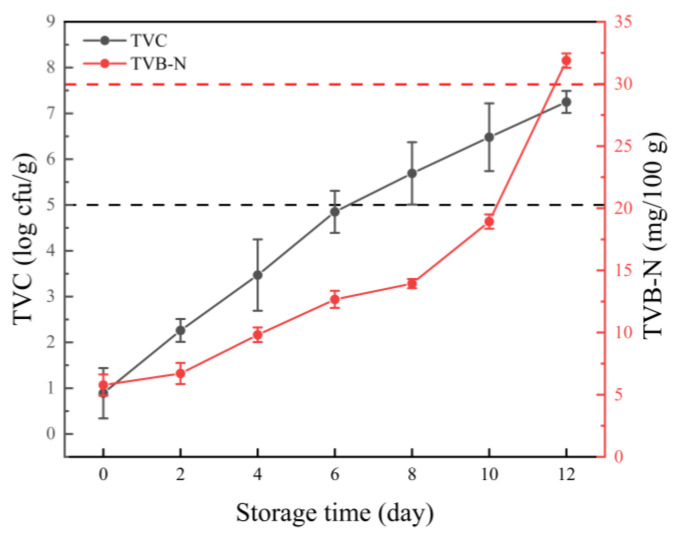
Changes in the TVC and TVB-N values of the boiled crayfish tails during 12 days of storage at 4 °C. The black dotted line indicates the maximum acceptable level of TVC in aquatic products, being 5.0 log CFU/g, and the red dotted line indicates the maximum acceptable level of TVB-N content in aquatic products, being 30 mg/100 g.

The changes in the TVC of the boiled crayfish tails during 12 days of storage are shown in Figure 1. The initial TVC value was lower than the detection limit (1.0 log cfu/g), implying that most of the bacteria were inactivated by boiling for 5 min. The growth of the bacteria in the tail meat steadily increased during storage. According to the *China National Food Safety Standard* (Animal Derived Aquatic Products; GB 10136–2015), the bacterial spoilage level is usually 5 log cfu/g in cooked aquatic products. As shown in Figure 1, the TVC value exceeded the acceptable limit on the eighth day, and it reached 7.25 log cfu/g at the end of storage.

TVB-N has been considered as an important spoilage indicator for fish, crustaceans, and other aquatic products. TVB-N represents unwanted nitrogen-containing compounds resulting from the degradation of food proteins, such as trimethylamine oxide (TMAO), trimethylamine (TMA), dimethylacetamide (DMA), and formaldehyde [29]. As shown in Figure 1, the TVB-N values continuously increased during refrigerated storage. The TVB-N values of the samples increased slowly in the first 4 days; however, a sharp rise was observed after 8 days of storage. Generally, a TVB-N value of 30 mg/100 g is considered to be the spoilage level and the inedible borderline [30]. Until day 12, the TVB-N values (31.88 mg/100 g) exceeded the acceptable limit. The crayfish tails exhibited visible spoilage and generated noticeable off-odors. Compared with the growth of the bacteria, the increases in the TVB-N values were accompanied by a lag phase which was likely due to the delay in the adaptation and growth of the bacteria that had undergone metabolism and had degraded protein to yield volatile basic nitrogen compounds [29].

### 3.2. HTS Analysis of the Crayfish Tails

#### 3.2.1. Illumina MiSeq Sequencing Analysis

In total, 79,728, 79,955, 80,152, and 79,989 raw reads were obtained for the raw, D0, D4, and D12 groups, respectively (Table 1). Afterward, quality filtering was carried out and the chloroplast sequences were removed. Thus, the raw reads were reduced to 79,582, 79,792, 80,020, and 79,883 clean reads, respectively. Then, chimeral filtration and quality control were performed. As a result, 72,523, 70,800, 70,624, and 54,269 effective tags for the raw, D0, D4, and D12 groups were achieved, respectively. Finally, this study determined the OTUs, as shown in Table 1, which were represented after processing and clustering. Among all the analyzed groups, the D0 group exhibited the lowest OTU value (104) while the raw group exhibited the highest OTU value (1110).

#### 3.2.2. Alpha Diversity Indices in the Samples

**Figure 2:**
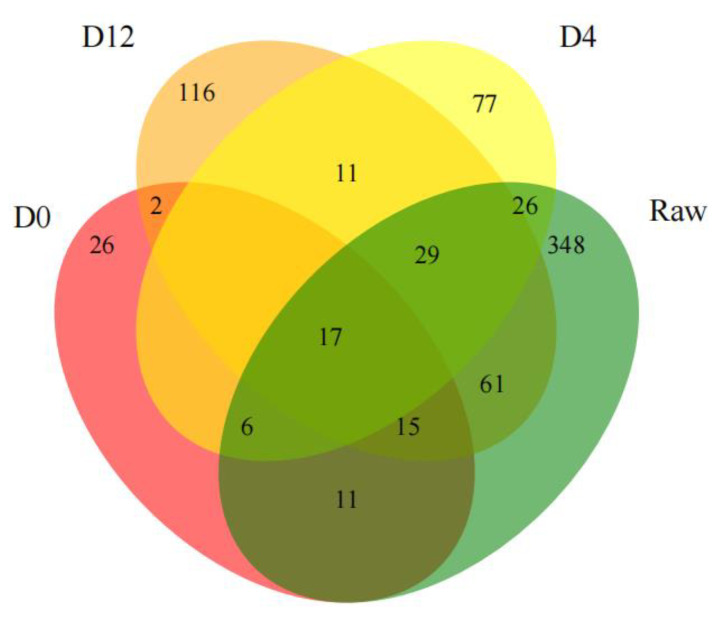
Venn diagram showing the unique and shared species in the bacterial communities in the different crayfish tail groups. Each circle in the figure represented one group. The number of circles overlapping areas represented the number of species shared between the groups. The numbers without overlapping area represented the number of unique species of the group.

Regarding the distribution of the bacterial species, a Venn diagram was constructed to analyze the similarities and differences in the bacterial diversity among the different groups. As shown in Figure 2, there were 17 species in all the groups throughout the storage period. Additionally, the number of unique species decreased from 496 (raw) to 60 (D0) after boiling, but it increased to 149 at the fourth day and 234 at the twelfth day. This indicated that boiling did not kill all the bacteria and that the number of bacterial species increased significantly with the increases in refrigeration time.

The results of the alpha diversity indices are shown in Table 2. The Chao1 index could be used to gauge species richness, i.e., how many species were present, and the Shannon and Simpson indices are commonly used to assess biological diversity, which is influenced by community richness and evenness. The highest of these three indices was the raw group, followed by the D4 group, the D12 group, and, lastly, the D0 group. Compared to the raw group, the decreased indices of the D0 group may have been due to the processing procedures, such as washing and boiling, which could have reduced the bacterial levels in the crayfish tails. Then, the nutrient-rich and suitable growth environment led to exacerbated bacterial growth, resulting in the higher indices in the D4 group. The reductions in bacterial richness and diversity at the end of storage (for D12) could be attributed to the nutrient deficiency, oxygen depletion, inhibited bacterial growth, and growing competition among the spoilage bacteria [31]. The coverage indices of all the groups were >99.9%, which reflected the efficacy and accuracy of the results in detecting nearly all bacteria in the groups.

#### 3.2.3. Effects of Storage Time on Bacterial Composition

##### Biodiversity and Relative Abundance of Phyla

**Figure 3:**
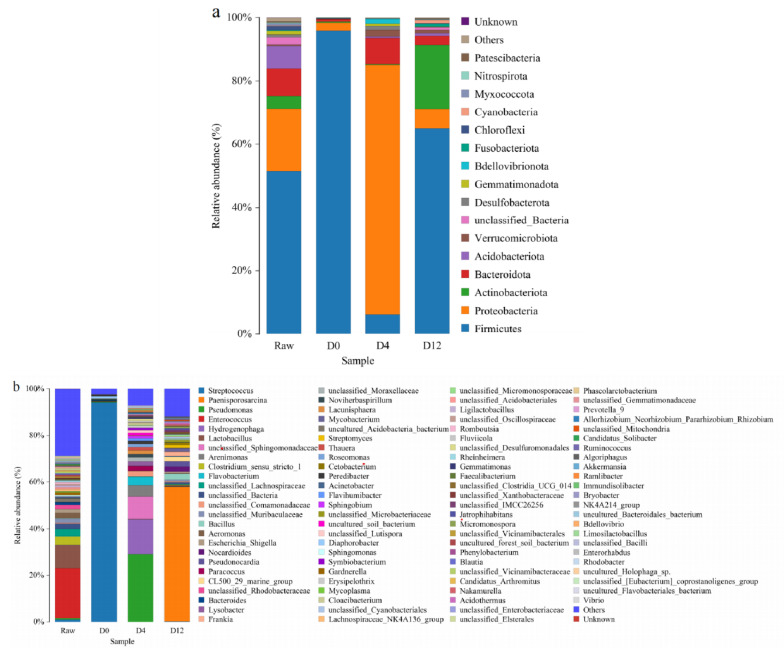
Bacterial community structure variation during the different stages. The relative abundances of the species of bacteria at the (**a**) phylum and (**b**) genus levels are shown. Each bar represents the relative abundance of each sample. Each color represents a particular phylum or genus.

The changes in the relative abundances at the phylum level within the bacterial communities of each group can be seen in Figure 3a. In total, 30 different phyla were observed in all groups. The most abundant bacterial species were found in the raw group, dominated by Firmicutes (51.56%) and Proteobacteria (19.73%), followed by Bacteroidota (8.58%), Acidobacteriota (7.19%), and Actinbacteriota (3.99%). These are often found in common aquatic products. Yan et al. [32] observed that the major phyla in the tail meat and hepatopancreas of red swamp crayfish during refrigerated storage were Proteobacteria, Bacteroidetes, and Firmicutes. Bekaert et al. [11] identified Proteobacteria and Actinbacteriota as the predominant microbiota in Norwegian lobster (*Nephrops norvegicus*) using PCR-DGGE technology. The richest bacteria in the raw group may have been related to the complex bacteria in the habitat and the pollution from the catching and transporting processes [32]. In the succession of bacterial communities, Firmicutes (64.96%) and Actinobacteriota (20.19%) were predominant in the D12 group. The bacteria from Firmicutes had been playing an important role during the whole storage period as they are Gram-positive and have the ability to resist dehydration as well as grow in extreme environments. Moreover, the relative abundance of Proteobacteria decreased and while that of Actinobacteriota increased in the D12 group compared to the D4 group. This may have been due to the inhibition of the growth of Proteobacteria by SSOs during the spoilage process. Most spoilage bacteria belong to Actinobacteriota and are associated with animal protein metabolism, and they cause off-odors in food products.

##### Biodiversity and Relative Abundance of Genera

The stacked-plot in Figure 3b shows the changes in bacteria genera during refrigerated storage. During spoilage, obvious differences in the bacterial communities were observed and presumed to be related to bacterial metabolism, competitive bacterial growth, and nutrient depletion [31]. For the raw crayfish tails, the dominant genera were *Enterococcus* (21.23%), *Lactobacillus* (9.73%), and *Aeromonas* (2.21%), which are frequently associated with seafood spoilage [17,33,34]. Current research has indicated that *Enterococcus* is negatively correlated with lipid oxidation products such as malondialdehyde (MDA), 4-hydroxy-2-nonenal (HNE), and 4-hydroxy-2-hexenal (HHE) [35]. The bacterium *Aeromonas* is a familiar conditional pathogen that can be responsible for human enteritis and septicemia. In addition, *Aeromonas* has various virulence factors such as adhesins, cytotoxins, enterotoxins, haemagglutinins, and hemolysins. At the beginning of storage (D0), the dominant genus was *Streptococcus* (94.13%), which belongs to the phylum Firmicutes. Some strains of *Streptococcus* can be important pathogens or conditionally pathogenic bacteria in seafood products [36]. Specific aldehydes produced during spoilage have been reported to inhibit the growth of *Streptococcus*, perhaps explaining the decrease in *Streptococcus* late in storage [35]. At day 4, *Pseudomonas* (29.00%), *Hydrogenophaga* (15.02%), *Arenimonas* (4.68%), and *Flavobacterium* (3.16%) became the dominant bacteria of the boiled crayfish tails. *Pseudomonas* is one of the most important spoilage bacteria in frozen aquatic products [33]. *Pseudomonas* is a psychrophilic Gram-negative bacterium with a wide variety and different functions, and it has the characteristics of fast proliferation and strong corruption. Under aerobically refrigerated storage, *Pseudomonas* could produce proteases and lipases that could potentially destroy the nutrients in food products, leading to food spoilage [37]. *Pseudomonas* and *Flavobacterium* have been proven as common bacteria of aquatic products from temperate waters [38]. At the end of storage (D12), the dominant genera in the boiled crayfish tails were *Paenisporosarcina* (57.69%), *Bacillus* (2.44%), *Nocardioides* (2.22%), *Frankia* (1.79%), *Mycobacterium* (1.48%), and *Streptomyces* (1.37%). *Paenisporosarcina* are spore-forming Gram-positive, aerobic or facultative anaerobic organisms. In addition, due to their strong resistance and rapid reactivation characteristics, *Paenisporosarcina* could be more competitive than other bacteria. Therefore, *Paenisporosarcina* was the dominant genus at the end of storage. *Bacillus* has been reported to produce diverse pyrazines which are nitrogenous flavor compounds [39]. The HTS results for the crayfish tails differed from those in previous studies [11], and this may have been influenced by the crayfish culture environment, the laboratory setting, and the experimenter’s method of operation.

#### 3.2.4. Clustering Heat Map of OTUs

**Figure 4:**
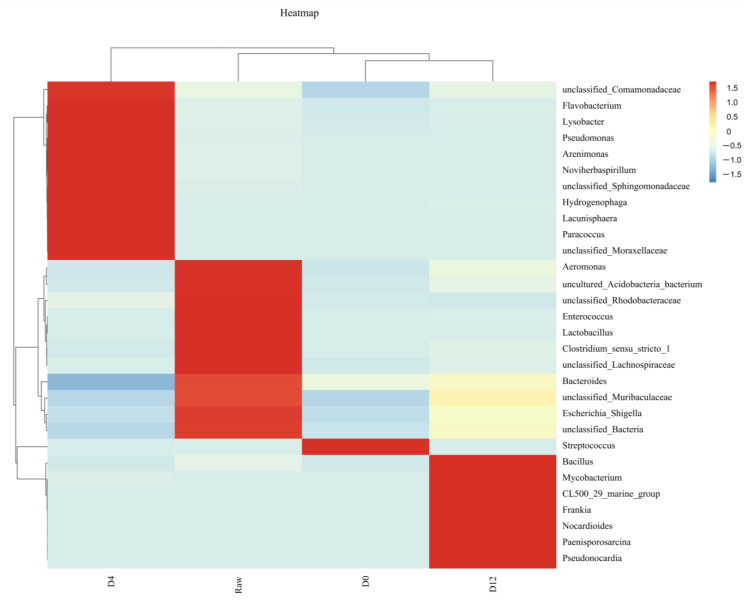
Heat map representing the genus abundances in the bacterial communities based on the 16S rRNA amplicon sequencing of the different crayfish tail groups.

Figure 4 shows the cluster analysis results of the raw crayfish tails and the boiled crayfish tails that had been stored at 4 °C for 0, 4, and 12 days. A two-dimensional heat map was plotted using the OTU data for the top 30 bacteria genera, with each column representing a group and each row indicating an OTU. In the heat map, the redder and the bluer colors represent the higher and the lower relative abundances, respectively. The heat map shows that significant differences existed in the bacterial flora in the four groups. The proportions of genera in the D4 group, including *Flavobacterium*, *Lysobacter*, *Pseudomonas*, *Arenimonas*, *Noviherbaspirillum*, *Hydrogenophaga*, *Lacunisphaera,* and *Paracoccus,* considerably decreased after refrigerated storage for 12 days. At the end of storage, the dominant genera in the boiled crayfish tails were *Bacillus*, *Mycobacterium*, *Frankia*, *Nocardioides*, *Paenisporosarcina,* and *Pseudonocardia*. This was potentially due to these bacteria being cold-tolerant as they can grow in low-temperature (<7 °C) conditions [40,41]. In addition, the dominant bacteria in the D12 group mainly included spore-forming Gram-positive Bacilli class bacteria. It has been reported that spore-forming Gram-positive bacteria have the highest pressure resistance, followed by non-spore-forming Gram-positive and Gram-negative bacteria [42,43]. According to previous research, *Bacillus* is the predominant bacteria in freshwater clams treated with high pressure [18]. Furthermore, *Bacillus* has been commonly detected in the intestinal tracts of crustaceans [44]. There is a possibility that the spoilage of the products was due to the germination and growth of spores of *Bacillus*. The spoilage was characterized by a soft and sticky texture, a loss of color, and a strong odor [45]. *Bacillus* was present in a high percentage at the end of the storage period; however, it was not detected during the remaining storage period, which may have been due to the time and the specific environment required for spore germination. During the current study, the main bacterial communities were identified at different points of the storage period, and this showed that the bacteria changed significantly over time. However, the metabolic abilities of specific taxa and their growth interactions were not explored. In order to clarify the relative contribution of each taxon to spoilage, the dominant spoilage bacteria were screened and identified using the culture-dependent methods, and the enzyme activities and spoilage potentials of the spoilage bacteria were determined.

### 3.3. Microbiological Analyses Based on the Culture-Dependent Methods

#### 3.3.1. Enumeration of Microorganisms

Viable counts of the different microbial groups from the deteriorating crayfish tails are shown in Table 3. At the end of storage, all the bacterial groups had reached high levels (between 6.72 log cfu/g (*Enterobacteriaceae*) and 7.70 log cfu/g (*Pseudomonads*)). *Pseudomonas* have been found to be the main spoilage bacteria that cause spoilage to degrade lipids and hydrolyze proteins [16,42,46,47]. Nevertheless, not all the bacteria present on the spoiled samples were involved in spoilage [40,48] as they may not have had the ability to spoil. Therefore, the abilities of the isolated strains to cause spoilage in boiled crayfish tails requires further investigation.

#### 3.3.2. Identification of the Isolated Strains

A total of 16 strains were isolated from the spoiled crayfish tails, and they differed in appearance, color, shape, and transparency (Table S1). These isolated colonies were identified by 16S rRNA gene sequencing. Each isolate exhibited one unique band in their denaturing gradient gel electrophoresis (DGGE) profiles. The relative identifications obtained in alignment with the NCBI and accession numbers for the submitted sequences are shown in Table 4. According to the 16S rRNA sequencing results, the 16 isolates belonged to 7 bacterial species which already had >99% similar sequences in the NCBI database. Among them, seven isolates (B, C, G, J, N, M, and P) were identified as *Acinetobacter lwoffii*, three isolates (E, F, and L) were identified as *Aeromonas veronii*, and two isolates (I and K) were identified as *Kurthia gibsonii*. Further, the strains A, D, H, and O were identified as *Pseudomonas sp.*, *Exiguobacterium aurantiacum*, *Lelliottia amnigena,* and *Citrobacter freundii*, respectively. In the current study, 43.75% of the isolates were identified as belonging to the genus *Acinetobacter*, which are typically isolated from putrefactive meat and seafood products [16,49]. Previous studies have suggested that *Acinetobacter* might lack proteolytic activity [50], but it could promote the growth of other spoilage bacteria through quorum-sensing signal molecules. Meanwhile, *Acinetobacter* has the potential to produce storage lipids such as wax esters (WEs) and triacylglycerols (TAGs) [51] and can thrive in poor nutrition conditions because of its good biofilm formation ability [52]. Moreover, *Aeromonas* and *Pseudomonas* are common bacteria in spoiled crustacean aquatic products [16,32]. *Aeromonas* has been reported to produce large amounts of putrescine and cadaverine, resulting in promoting the active formation of TVB-N during refrigerated storage [53]. In general, *Pseudomonas* has mainly been observed at aerobically low temperatures conditions, possessing a high genetic diversity and metabolic versatility [54,55]. Pseudomonas is a specific spoilage bacterium that produces unpleasant odors due to the production of ammonia, H_2_S, and TMA [56]. *Citrobacter freudii* was also isolated from spoiled crayfish by Chen et al. [57]. *Citrobacter* is a Gram-negative facultative anaerobic bacterium which has frequently been found in the intestinal tracts of aquatic products and may induce disease in aquatic animals. *Lelliottia amnigena* and *Citrobacter freundii* belong to the family Enterobacteriaceae, which has been reported to be involved in the accumulation of biogenic amines [58].

#### 3.3.3. Enzymatic Characteristics of the Selected Isolates

**Figure 5:**
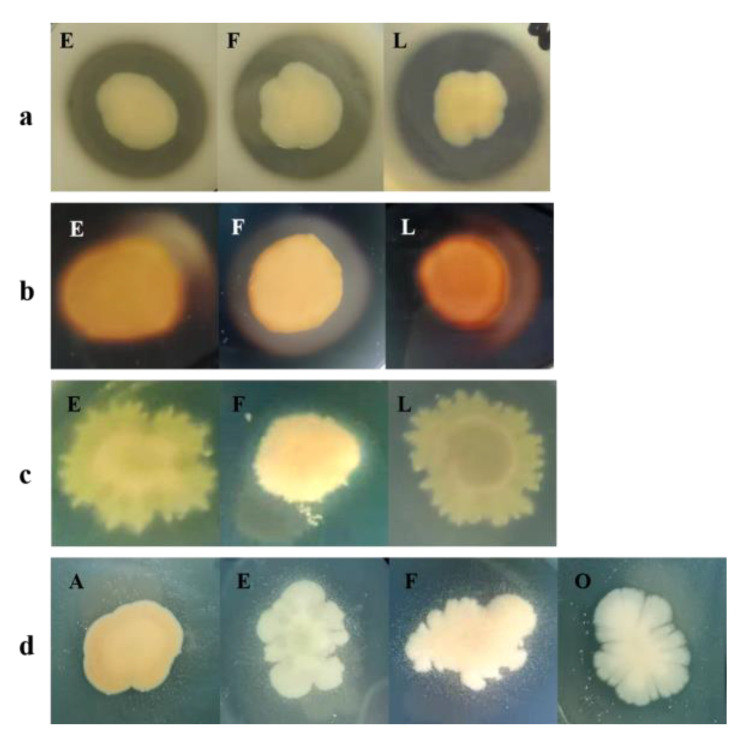
Enzymatic activities of the bacterial strains. (**a**) Detection of proteolytic activity. (**b**) Detection of amylolytic activity. (**c**) Detection of phosphorolytic activity. (**d**) Detection of lipolytic activity.

Enzymes are one of the key factors resulting in defects related to food odors and shelf life issues during refrigerated storage [59]. Using agar diffusion assays, all isolates were evaluated for their ability to produce protease, lipase, phospholipase, and amylase (Figure 5). The isolates of the genus *Aeromonas* (strains E, F, and L) showed proteolytic, lipolytic, phosphorolytic, and amylolytic activities, though strain L had no lipolytic activity. The *Pseudomonas* isolate (strain A) and *Citrobacter* isolate (strain O) also showed lipolytic activity. In a previous report, *Aeromonas* showed strong proteolytic activity and *Enterobacter* had a tendency to produce lipases [60], and these results were similar to the results of this study. *Pseudomonas* is often mentioned for its enzymatic activity [61]. There is some evidence that proteases have the ability to degrade protein and yield free amino acids, which leads to a loss of freshness [29]. The unpleasant smell found in spoiled samples is associated with the rendition of lipases and, thus, lipid oxidation [62,63]. In addition, phospholipases can enhance lipolysis by degrading various components [60]. Amylases could promote the metabolism of bacteria and destroy the structure of carbohydrates. Consequently, the strains E, F, L, A, and O from the genera *Aeromonas*, *Pseudomonas,* and *Citrobacter* could have significantly contributed to the spoilage of the boiled crayfish tails and be regarded as the SSOs of the boiled crayfish tails.

#### 3.3.4. Spoilage Potential of the SSOs

**Figure 6:**
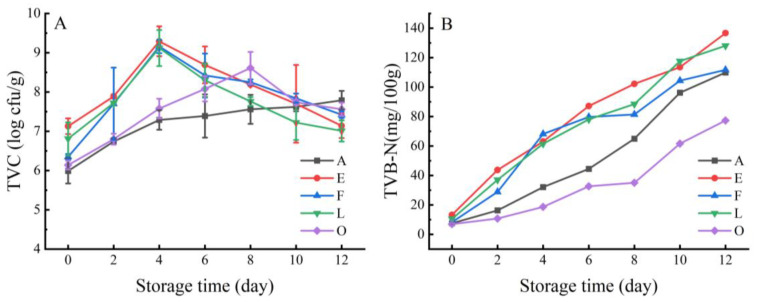
Development of (**A**) TVC, log cfu/g, and (**B**) TVB-N, mg/100 g, in the sterile boiled crayfish tails inoculated with the different bacterial groups during storage for 12 days at 4 °C.

We further characterized the spoilage potentials of the spoilage bacteria by more precisely monitoring the growth and chemical changes in the spoilage bacteria. The five SSOs (strains A, E, F, L, and O) were separately inoculated into sterile boiled crayfish tails. The spoilage ability of each strain on the crayfish tails was determined based on the TVC and TVB-N values during 12 days of refrigerated storage (Figure 6). The inoculated level of each strain was approximately 6.0–7.0 log cfu/g counts. The strain E, F, and L (the isolates of *Aeromonas*) groups grew rapidly and reached their maximum growth levels (above 9.0 log cfu/g) on day 4, and then they decreased slowly. No significant differences were observed in the maximum TVC values of the inoculated groups with the strains E, F, and L. A smooth growth in TVC values was observed for strain O (the *Citrobacter* isolate) during storage, with a maximal level of 8.61 log cfu/g on day 8. The viable counts of the strain A (*Pseudomonas* isolate) group increased progressively and reached 7.79 log cfu/g at the end of storage.

After inoculation, the initial TVB-N values of all the samples were 7.0–14.0 mg/100 g, and they increased gradually during refrigerated storage (12 days). The samples inoculated with strain E showed the maximum production of TVB-N among all the tested samples, followed by the groups inoculated with strains L, F, and A. At the end of storage, there were no significant differences in the TVB-N values between the groups inoculated with strain F and strain A. The TVB-N values of the group inoculated with strain E grew slowly in the early stages (days 0–8), and they were lowest at the end of storage. These spoilage bacteria have the ability to produce metabolites such as TVB-N and cause the formation of spoilage off-flavors [9,48,64]. According to the TVC and TVB-N values, strains E, F, and L (*Aeromonas*) exhibited stronger spoilage abilities than the rest of the strains in the boiled crayfish tails throughout refrigerated storage. This was similar to the findings of Stohr et al. [65], who found that a strain of *Aeromonas* with strong spoilage ability produced a large amount of TVB-N when inoculated on sterile aquatic products.

## 4. Conclusions

In this study, the bacterial community diversity and dynamics in crayfish tails during refrigerated storage were analyzed by using culture-independent methods. As a result of the HTS, 30 different phyla and 100 different genera were identified in all the groups. With increasing storage time, the HTS results showed that the community richness increased significantly. For the different storage periods, the dominant bacterial communities were significantly different. *Streptococcus* became the only dominant bacteria at the beginning of storage, *Pseudomonas* became the dominant bacteria at the storage metaphase, and *Paenisporosarcina* became an overwhelming dominant genus at the end of storage. Furthermore, 16 isolates were identified at the end of storage by culture-dependent methods. All 16 isolates were identified genera species that could be detected in the HTS results of the groups on day 12. The three isolates identified as *Aeromonas* were found to have the highest spoilage capacities. Consequently, detecting and analyzing the bacterial compositions in crayfish tails under refrigeration can provide a theoretical basis for determining the quality and safety of the products. There is a possibility that this study may provide a fresh perspective on the spoilage process of refrigerated crayfish tails, and this may lead to the development of new preservation methods and sterilization techniques in the future.

## Figures and Tables

**Table 1:** Illumina MiSeq sequencing basic information.

Samples	Raw Reads	Clean Reads	Effective Tags	OTUs
Raw	79,728	79,582	72,523	1110
D0	79,955	79,792	70,800	104
D4	80,152	80,020	70,624	252
D12	79,989	79,883	54,269	436

**Table 2:** Alpha diversity indices of the groups.

Samples	Chao1	Shannon	Simpson	Coverage
Raw	1110.00	0.9751	8.0722	>99.9%
D0	104.00	0.6590	2.1099	>99.9%
D4	252.00	0.9514	5.6186	>99.9%
D12	436.00	0.7578	4.6925	>99.9%

**Table 3:** Changes in the bacterial counts at the end of refrigerated storage.

Microbiological Indices	Viable Count (log cfu/g)
TVC	7.85 ± 0.04 ^b^
LAB	7.51 ± 0.39 ^b^
Enterobacteriaceae	6.72 ± 0.10 ^a^
Pseudomonads	7.70 ± 0.06 ^b^
Molds and yeasts	7.34 ± 0.51 ^ab^
TPC	7.69 ± 0.02 ^b^

Note: The figures in the table are means and standard errors. ^a^ and ^b^ within the rows refer to significant differences (*p* < 0.05).

**Table 4:** Strains identified by means of the 16S rDNA sequencing fragments from the pure cultures isolated from the spoiled boiled crayfish tails.

Isolate Number	Closest Relatives	Per. Ident	Accession No.
A	*Pseudomonas* sp.	99.86%	KY942195.1
B	*Acinetobacter lwoffii*	99.46%	KU977288.1
C	*Acinetobacter lwoffii*	99.53%	CP054822.1
D	*Exiguobacterium aurantiacum*	99.93%	CP101462.1
E	*Aeromonas veronii*	99.76%	CP034967.1
F	*Aeromonas veronii*	99.85%	KY867531.1
G	*Acinetobacter lwoffii*	99.86%	CP054822.1
H	*Lelliottia amnigena*	99.93%	LR134135.1
I	*Kurthia gibsonii*	99.80%	AM118426.1
J	*Acinetobacter lwoffii*	99.73%	CP019143.2
K	*Kurthia gibsonii*	99.80%	KJ872770.1
L	*Aeromonas veronii*	99.73%	EU488695.1
M	*Acinetobacter lwoffii*	99.78%	EU779994.1
N	*Acinetobacter lwoffii*	99.63%	MW433857.1
O	*Citrobacter freundi*	99.46%	CP044098.1
P	*Acinetobacter lwoffii*	99.73%	KF228924.1

## Data Availability

The data are contained within the article.

## Supplementary Materials

The following supporting information can be downloaded at: https://www.mdpi.com/article/10.3390/foods12163006/s1, Table S1: Description of the morphological characteristics of the isolated bacterial colonies.
