# Supplementary material for: Bacterial Changes in Boiled Crayfish between Different Storage Periods and Characterizations of the Specific Spoilage Bacteria
Source: Foods. 2023 Aug 9;12(16):3006. doi: 10.3390/foods12163006 (PMC10453257; doi:10.3390/foods12163006)
Supplement: Supplementary file 1 [file foods-12-03006-s001.zip › foods-2516540-supplementary.pdf]

foods

Table S1. Description of morphological characteristics of the isolated bacterial colonies.

Number	Appearance	Colour	Shape	Transparency
A	smooth, small, raised entire edges	pale yellow	circular	non-transparent
B	smooth, small,elevated centre	white	non-circular, corrugated edges	non-transparent
C	smooth, small, raised entire edges	pale yellow	circular	non-transparent
D	smooth, small, elevated centre	yellow	non-circular, corrugated edges	non-transparent
E	smooth, large, raised entire edges	pale yellow	non-circular, corrugated edges	translucent
F	smooth, large, raised entire edges	white	non-circular, corrugated edges	translucent
G	smooth, small, raised entire edges	white	circular	non-transparent
H	smooth, small, depression centre	pale yellow	circular	non-transparent
I	smooth	yellow	irregular	non-transparent
J	smooth, small, elevated centre	white	circular	non-transparent
K	smooth	yellow	irregular	non-transparent
L	smooth, large, raised entire edges	yellow	circular	non-transparent
M	smooth, small, raised entire edges	pale yellow	circular	non-transparent
N	smooth, small, raised entire edges	white	circular	non-transparent
O	smooth, small, depression centre	pale yellow	circular	non-transparent
P	smooth, small, raised entire edges	white	circular	translucent
